# The mediating role of dietary factors and leisure time physical activity on socioeconomic inequalities in body mass index among Australian adults

**DOI:** 10.1186/1471-2458-13-1214

**Published:** 2013-12-21

**Authors:** Emma Gearon, Kathryn Backholer, Allison Hodge, Anna Peeters

**Affiliations:** 1 Obesity and population health unit, Baker IDI Heart and Diabetes Institute, Melbourne, Australia; 2 Cancer Epidemiology Centre, Cancer Council Victoria, Melbourne, Australia

**Keywords:** Obesity, Socioeconomic factors, Socioeconomic inequalities, Food and beverages, Leisure activity, Diet, Physical activity, Epidemiologic methods

## Abstract

**Background:**

The relationship between socioeconomic position and obesity has been clearly established, however, the extent to which specific behavioural factors mediate this relationship is less clear. This study aimed to ascertain the contribution of specific dietary elements and leisure-time physical activity (LTPA) to variations in obesity with education in the baseline (1990–1994) Melbourne Collaborative Cohort Study (MCCS).

**Methods:**

18, 489 women and 12, 141 men were included in this cross-sectional analysis. A series of linear regression models were used in accordance with the products of coefficients method to examine the mediating role of alcohol, soft drink (regular and diet), snacks (healthy and sweet), savoury items (healthy and unhealthy), meeting fruit and vegetable guidelines and LTPA on the relationship between education and body mass index (BMI).

**Results:**

Compared to those with lowest educational attainment, those with the highest educational attainment had a 1 kg/m^2^ lower BMI. Among men and women, 27% and 48%, respectively, of this disparity was attributable to differences in LTPA and diet. Unhealthy savoury item consumption and LTPA contributed most to the mediated effects for men and women. Alcohol and diet soft drink were additionally important mediators for women.

**Conclusions:**

Diet and LTPA are potentially modifiable behavioural risk factors for the development of obesity that contribute substantially to inequalities in BMI. Our findings highlight the importance of specific behaviours which may be useful to the implementation of effective, targeted public policy to reduce socioeconomic inequalities in obesity.

## Background

In developed countries, the prevalence of obesity is socially patterned whereby those from a lower socioeconomic position (SEP) are more likely to be obese than their higher SEP counterparts [1]. These trends are clearer and more consistent for women than men [2]. Because obesity is associated with an increased risk of morbidity and mortality, this is likely to lead to a further disproportionate burden of ill-health among the most disadvantaged [3].

In order to reduce socioeconomic inequalities in obesity prevalence, it is essential that obesity prevention interventions and policies are targeted at specific health behaviours and environments that contribute to the observed inequalities. These factors are likely to be multifactorial and complex [4], however, diet and physical activity, two modifiable behavioural risk factors for the development of obesity, are likely to be important. While it is well established that diet and physical activity are socially patterned [5-8] and influence energy balance [4,9], the extent to which specific dietary factors and physical activity mediate the relationship between SEP and obesity remains largely unknown.

Only a small number of studies have examined the mediating role of modifiable health behaviours on the relationship between SEP and adiposity. Using a range of SEP indicators, combinations of diet and physical activity have been found to explain 25% [10] and 27% [11] of the disparity in obesity prevalence between high and low SEP groups among men, and 18%, 40% [12] and 45% [11] among women. However, these previous studies are generally limited by the paucity of dietary variables available, their inability to account for the mediating role of specific diet or physical activity factors [10,11], or by limitations associated with their study population [8,12,13].

The aim of the current study was to determine the extent to which leisure time physical activity (LTPA) and a range of specific dietary factors mediate the relationship between education, as a measure of SEP, and body mass index (BMI) among men and women. To do this we used cross-sectional data from 12,141 men and 18,489 women in the baseline survey of the Melbourne Collaborative Cohort Study (MCCS).

## Methods

### Study design and participants

Data from the baseline survey of the MCCS was used for this cross sectional analysis. Subjects were volunteers from Melbourne who were recruited from the community between 1990 and 1994 using electoral rolls, telephone books, advertisements and community announcements [12]. To explore a wide range of genetic and lifestyle factors, the study deliberately oversampled migrants from Southern Europe, who comprised a quarter of the 41,514 individuals recruited into the study.

Data was collected by trained interviewers, and included a questionnaire in the participant’s preferred language, a food frequency questionnaire (FFQ), and physical measurements. The questionnaire asked for information on the participant’s demographic, lifestyle and medical history, including smoking and drinking status, LTPA, country of birth and education. The physical measurements included height and weight. For the current analysis, subjects were excluded if they were Southern European born (n = 9974) due to the strong correlation between educational attainment and country of birth in the sample (Southern European born participants comprised 84.7% of the lowest education category and 4% of the highest education category). Subjects were additionally excluded if they were missing data for any exposure, mediator or outcome variable (n = 82) or had an implausibly high or low energy intake (in the top or bottom 1% of the entire cohort [14]) (n = 828), resulting in a final study sample size of 18,489 women and 12,141 men.

### Data collection

Highest educational attainment was the socioeconomic indicator used for this analysis. Education was ascertained from the question ‘What is the highest level of education you completed?’ and dichotomised into those who had completed high school (high education group) and those who had not (low education group).

Information on participants usual diet over the previous twelve months was captured using a FFQ developed specifically for the cohort [15] and verified in a sub-sample of the population [15]. For this sub-sample of 800 men and women of similar demographic background to cohort participants, the validation of the FFQ included 121 food items identified from weighed food records. Nine possible frequency options, ranging from “never or less than once a month” to “six or more times per day” were available. Food items included in the current analysis were converted into a continuous measure of times per week. To limit the number of food elements entered into our regression models, we first created food groups that represented a broad range of nutritional profiles. These included ‘healthy snacks’, ‘healthy savoury items’, ‘fruit and vegetables’, ‘sweet snacks’ and ‘unhealthy savoury items’. Soft drink, diet soft drink and alcohol were additionally included as separate variables. The selection of food items to include in the groups was informed by a recent review on the socioeconomic patterning of diet [5] and a recent pooled cohort study of 120,877 U.S. women and men investigating the association of changes in dietary habits and long-term weight gain [9] (see Table 1 for details of dietary elements). LTPA was derived from three questions asking participants how frequently (times per week) they walked for recreation or exercise and did vigorous or non-vigorous activities in their leisure time in the 6 months prior to the questionnaire. The physical activity score was then derived by combining the frequency of the three activity types (where vigorous activity was given double weighting) and each participant was assigned a physical activity score between 0 and 16 as described elsewhere [16]. LTPA will be used in reference to the physical activity score throughout this paper.

**Table 1:** **Composition of variables used for analysis in the MCCS**^**^ **^**study population**

**Behaviour**	**Components**	**Units**
LTPA*	Physical activity score (walking, non-vigorous, vigorous)	Times/week
Alcohol		g/day
Diet soft drink		Times/week
Soft drink		Times/week
Healthy snacks	Yoghurt	0 = Consume nuts and yoghurt
		< 0.5 times/week
	Nuts	1 = Consume nuts *or* yoghurt 0.5 ≥ times/week
Healthy savoury items	Wholemeal bread, rolls, toast	Continuous sum of each variable in times/week
	Wheat germ	
	Muesli	
	Chicken, boiled or steamed	
	Fish, boiled, steamed or baked	
Fruit and vegetables (meeting guidelines)	Fruit	0 = Consume fruit < 2 times/day or veg < 5 times/day
	Vegetable (except potato)	1 = Consume fruit ≥ 2 times/day *and* veg ≥5 times/day
Sweet snacks	Cakes and sweet pastries	Continuous sum of each variable in times/week
	Confectionary	
	Chocolate	
	Sweet biscuits	
Unhealthy savoury items	White bread	Continuous sum of each variable in times/week
	Pies and savoury pastries	
	Dim-sims and spring rolls	
	Pizza	
	Corn and potato chips	
	Roast or fried chicken	
	Roast or fried potatoes	
	Sausages or frankfurters	
	Salami or continental sausages	
	Manufactured luncheon meats	
	Corned beef	

* Leisure time physical activity (times/week).

^^^ This study utilises a sample of 30,630 participants from the baseline sample of the Melbourne Collaborative Cohort Study (MCCS) which was conducted in Melbourne between 1990 and 1994.

Participants were asked if they were never smokers, former smokers or current smokers. Self reported history of angina, stroke, heart attack, cancer and diabetes was also collected.

Trained interviewers measured height (with a wall-mounted stadiometer; cm) and weight (with a digital scale; kg). BMI was calculated as weight in kilograms divided by height in metres squared (kg/m^2^) and was used as a continuous measure.

### Statistical analyses

#### Descriptive statistics

Descriptive statistics were used to compare baseline characteristics across sex specific SEP groups and are presented as mean and standard deviation (sd) for normally distributed data or median and inter-quartile range (first quartile, third quartile (Q_1_,Q_3_)) for non-normally distributed variables.

#### Mediation analysis

To determine the mediating role of the dietary variables and LTPA on the relationship between education and BMI we used a series of linear regression models in accordance with the product of coefficients mediation method [17]. Education was used as the exposure, BMI as the outcome and the diet groups and LTPA were used as mediators. All models were adjusted for age and smoking status, and given the known sex differences in the socioeconomic distribution of BMI and obesity [18], all analyses we stratified by sex. The mediation method involved several steps as follows [19]: 1) the total relationship between education and BMI was determined (*c* coefficient); 2) the independent relationship between education and each mediator was determined in separate regression models (*a* coefficient); 3) the relationship between each mediator and BMI, adjusted for all other mediators and the exposure (education), were determined in a single regression model. This regression model yielded both the relationship between each mediator and BMI (*b* coefficient) and the relationship between education and BMI after controlling for all mediating variables (*c’* coefficient); 4) for each mediator that was significantly related to both education and BMI, the product of the *a* and *b* coefficients was derived to obtain the independent mediating effect of each mediator on the relationship between education and BMI. The sum of all indirect effects (sum of ab for all individual mediators) yielded the total indirect effect through all mediators; 5) the proportion mediated (for each individual mediator and for all mediators combined) was determined by dividing the indirect effect (ab coefficient) by the total effect (c coefficient).

All regressions were performed using a bootstrapping procedure, with 5000 replications, to obtain all coefficients and 95% confidence intervals. Significance in the analyses was set at the 5% level.

Stata version 11 was used to perform all analyses (Stata Corp. LP., College Station, TX, USA).

#### Sensitivity analysis

We performed a number of sensitivity analyses to test the robustness of our results. We tested the effect of 1) additionally adjusting for chronic disease and, among females adjusting for parity; 2) redefining SEP such that the high education group included only those who had completed tertiary education; 3) effect modification by smoking status by limiting the primary analysis to the population of never smokers.

### Ethics

The MCCS study protocol was approved by the Cancer Council Victoria’s Human Research Ethics Committee and subjects gave written consent to participate [20]. Ethics approval for the current study was obtained from Alfred Hospital Ethics Committee; Alfred ethics project number 55/12.

## Results

### Baseline characteristics

Participants with a lower educational attainment were generally older and were more likely to be current smokers, to have chronic disease (with the exception of cancer for women) and to have a higher BMI than those with a higher educational attainment, these patterns were similar for men and women (Table 2).

**Table 2:** **Baseline characteristics of the MCCS**^**^ **^**study population by education and sex**

	**LOW EDUCATION**		**HIGH EDUCATION**	
	**Mean (sd)/median (Q**_**1**_**,Q**_**3**_**)/%**		**Mean (sd)/median (Q**_**1**_**,Q**_**3**_**)/%**	
	**Men**	**Women**	**Men**	**Women**
n (% of total)	4,671 (15%)	10,109 (33%)	7,470 (24%)	8,380 (27%)
Body mass index (BMI)	27.3 (3.8)	26.5 (4.8)	26.3 (3.4)	25.3 (4.4)
Obese (%)	20.4%	19.6%	12.2%	13.1%
Age	57.4 (8.7)	56.8 (8.5)	54.1 (9.1)	52.9 (8.7)
Heart attack, stroke and/or angina (%)	11.8%	5.3%	7.6%	2.7%
Cancer (%)	10.5%	9.4%	7.9%	9.7%
Diabetes (%)	3.7%	2.4%	2.3%	1.4%
Current smokers (%)	14.9%	10.9%	9.2%	7.9%
Fruit and vegetables (%)^#^	16.2%	33.3%	23.9%	42.2%
Healthy snacks (%)^§^	56.2%	73.5%	73.%	86.2%
LTPA*	4 (1.5, 5.5)	4 (1.5, 5.5)	4 (1.5, 8)	4 (1.5, 7)
Alcohol (g/day)	10.4 (0.8, 27.8)	0.9 (0, 8.6)	12.9 (2.3, 28.5)	4.5 (0, 15.1)
Healthy savoury items*	6 (1, 14)	7.5 (3, 17.5)	8 (3.5, 17.5)	9.5 (5, 18.5)
Sweet snacks*	6.5 (2, 13)	6 (2, 12)	6 (2.5, 12)	5.5 (2, 10.5)
Unhealthy savoury*	10.5 (6, 20)	7 (3.5, 12.5)	9 (5, 14)	5.5 (3, 10)

^#^ (proportion meeting guidelines, consuming ≥ 2 fruits and ≥5 vegetables per day).

^§^ (proportion consuming nuts or yoghurt ≥ 0.5 times/week).

* Leisure time physical activity (times/week); sd standard deviation; Q_1_,Q_3_ (first quartile, third quartile).

^^^ This study utilises a sample of 30,630 participants from the baseline sample of the Melbourne Collaborative Cohort Study (MCCS) which was conducted in Melbourne between 1990 and 1994.

Those with a lower educational attainment appeared less likely to engage in high levels of LTPA or meet fruit and vegetable guidelines, and likely to consume less alcohol and healthy savoury items and to consume more unhealthy savoury items. These patterns were similar for both sexes. Consumption of diet soft drink and soft drink within this population was too low to compare the median intake. Over 70% of the population reported drinking diet soft drink less than once per month, and around half reported drinking soft drink less than once per month.

### Mediation results

#### The relationship between education and BMI (c coefficient)

We observed a negative relationship between educational attainment and BMI among men and women. Men with a lower education were found to have a BMI that was on average 0.89 kg/m^2^ (95% CI, 0.75 - 1.03) higher compared to those with a higher educational attainment. Among women the mean difference in BMI was 1.01 kg/m^2^ (95% CI 0.88 - 1.14).

#### The relationship between education and each mediator (a coefficient)

Among men, LTPA, the consumption of healthy snacks, the consumption of healthy savoury food items and meeting fruit and vegetables guidelines occurred more frequently in those who had higher educational attainment. Conversely, the consumption of diet soft drink, soft drink, sweet snacks and unhealthy savoury food items occurred more frequently in those of a lower educational attainment. Alcohol consumption frequency was not significantly associated with educational attainment among men in this study.Similarly, among women, although the magnitudes differed, the same patterns of association with education were observed for LTPA and dietary behaviours, with the exception of alcohol consumption, which was more common among women with higher educational attainment (see column one of Table 3).

**Table 3:** **The mediating role of diet and physical activity on the relationship between SEP and BMI using the product of coefficients mediation method in the MCCS**^**^ **^**study population**

		Association between education* and mediator		**Association between mediator and BMI†**		**Mediated effect**		**Proportion mediated**
		** a**	**95% CI**	** b**	**95% CI**	** ab**	**95% CI**	**%**
LTPA^#^	M	** 0.69**	**(0.55, 0.83)**	**−0.10**	**(−0.12, -0.09)**	**−0.07**	**(−0.09, -0.05)**	8%
	W	** 0.70**	**(0.59, 0.80)**	**−0.15**	**(−0.17, -0.13)**	**−0.10**	**(−0.12, -0.09)**	10%
Alcohol	M	0.35	(−0.58, 1.31)	0.00	(0.00, 0.01)			
	W	** 3.50**	**(3.11, 3.89)**	**−0.03**	**(−0.03, -0.02)**	**−0.10**	**(−0.13, -0.08)**	10%
Diet soft drink	M	**−0.23**	**(−0.37, -0.09)**	** 0.16**	**(0.13, 0.18)**	**−0.04**	**(−0.06, -0.01)**	4%
	W	**−0.41**	**(−0.52, -0.30)**	** 0.20**	**(0.18, 0.23)**	**−0.08**	**(−0.11, -0.06)**	8%
Soft drink	M	**−0.68**	**(−0.84, -0.52)**	** 0.06**	**(0.04, 0.08)**	**−0.04**	**(−0.06, -0.03)**	5%
	W	**−0.33**	**(−0.42, -0.25)**	** 0.05**	**(0.02, 0.08)**	**−0.02**	**(−0.03, -0.01)**	2%
Healthy snacks	M	** 0.14**	**(0.13, 0.16)**	−0.01	(−0.15, 0.13)			
	W	** 0.11**	**(0.10, 0.12)**	**−0.27**	**(−0.46, -0.09)**	**−0.03**	**(−0.05, -0.01)**	3%
Healthy savoury	M	** 1.90**	**(1.54, 2.25)**	**−0.02**	**(−0.03, -0.01)**	**−0.04**	**(−0.06, -0.03)**	4%
	W	** 2.42**	**(2.14, 2.71)**	**−0.01**	**(−0.02, 0.00)**	**−0.03**	**(−0.05, -0.01)**	3%
Fruit and vegetables	M	** 0.07**	**(0.06, 0.09)**	−0.12	(−0.27, 0.03)			
	W	** 0.09**	**(0.07, 0.10)**	0.12	(−0.02, 0.25)			
Sweet snacks	M	**−0.68**	**(−1.08, -0.29)**	**−0.03**	**(−0.03, -0.02)**	** 0.02**	**(0.01, 0.03)**	−2%
	W	**−1.16**	**(−1.46, -0.86)**	**−0.01**	**(−0.02, 0.00)**	** 0.01**	**(0.00, 0.02)**	−1%
Unhealthy savoury	M	**−2.66**	**(−3.04, -2.27)**	** 0.03**	**(0.02, 0.04)**	**−0.07**	**(−0.10, -0.05)**	8%
	W	**−2.26**	**(−2.5, -2.02)**	** 0.06**	**(0.05, 0.07)**	**−0.14**	**(−0.16, -0.11)**	13%
Total mediated effect	M					**−0.24**	**(−0.29, -0.20)**	27%
	W					**−0.49**	**(−0.54, -0.44)**	48%

*Note*: The total mediated effect and proportion mediated was calculated only for those variables which were significantly related to education (a) or BMI (b). Boldface is used to denote significant relationships. * Lowest educational attainment is the reference category.

† Where zero consumption or not meeting guidelines is the reference category.

^#^ Leisure time physical activity (times/week).

^^^ This study utilises a sample of 30,630 participants from the baseline sample of the Melbourne Collaborative Cohort Study (MCCS) which was conducted in Melbourne between 1990 and 1994.

#### The relationship between each mediator and BMI (b coefficient)

Among men, LTPA, the consumption of healthy savoury food items and the consumption of sweet snacks were associated with having a lower BMI, while the consumption of diet soft drink, soft drink and unhealthy savoury food items were associated with having a higher BMI. There was a suggestion of a positive association between alcohol consumption and BMI among men, while healthy snacks and meeting fruit and vegetable guidelines were not associated with BMI for men in this study.

Among women, similar patterns of association, but with different magnitudes, were observed for LTPA and dietary behaviours, with the exception of healthy snacks and alcohol consumption, which were associated with having a lower BMI (see column two of Table 3).

#### The mediating role of diet and LTPA on the relationship between education and BMI (ab coefficient)

LTPA and all dietary variables included in this analysis accounted for 0.24 kg/m^2^ (27%) and 0.49 kg/m^2^ (48%) of the difference in BMI observed between the two education groups for men and women, respectively.

The contributions of individual mediators are shown in Table 3. Meeting fruit and vegetable guidelines was the only dietary variable not associated with socioeconomic inequalities in BMI for men or women and the consumption of sweet snacks was inversely related to socioeconomic inequalities in BMI, accounting for (−2%) and (−1%) in men and women, respectively.

### Sensitivity analyses

#### Chronic disease, parity, altered education split and never smokers

No substantial changes to the overall mediated effect were observed when the analysis was adjusted for chronic disease, nor for parity, nor for when the high education category of SEP was defined as completion of tertiary education (results not shown), nor for when the analysis was conducted in the never smoking population, comprising 5559 men and 11915 women.

In light of the finding that sweet snacks had a positive mediating effect, two further sensitivity analyses were performed.

Firstly, the effect of removing all sweet snacks from the total mediated effect (though they remained as confounders in the calculations for other variables) was explored. The total mediating role of diet and LTPA in this scenario was 0.26 kg/m^2^ (29%) and 0.50 kg/m^2^ (49%) in men and women, respectively.

Additionally, based on evidence that specific sweet foods can differ in their relationship to socioeconomic status and BMI [5,9], the variables cake and sweet pastries, and sweet biscuits were removed from the sweet snacks group, allowing us to observe the independent mediating role of chocolate and confectionary. In this scenario, sweet snacks (chocolate and confectionary only) were no longer significantly related to SEP in men (0.07 95% CI (−0.23,0.15)), and were no longer significantly related to BMI in women (0.00 95% CI (−0.01,0.01)). However, the total mediated effect did not change substantially in either sex.

## Discussion

Using a cross-sectional analysis of 12,141 men and 18,489 women, we examined the association between educational attainment and BMI, and the extent to which this association was mediated by LTPA and a range of dietary factors. We observed an inverse association between educational attainment and BMI, whereby those with a lower educational attainment had a BMI approximately 1 kg/m^2^ higher than those with a higher educational attainment. Differences in diet and LTPA between education levels accounted for 27% and 48% of the socioeconomic disparity in BMI for men and women, respectively. Of particular importance for both sexes were lower levels of LTPA and a higher consumption of unhealthy savoury foods among those with a lower educational attainment. A higher consumption of alcohol and a lower consumption of diet soft drink among women with a higher education were also important mediators.

The magnitudes of the total mediated effects for men and women are similar to other existing studies examining the mediating role of health behaviours in the relationship between SEP and a measure of adiposity. Similar to our study, Kavanagh *et al.*, [11] investigated the role of health behaviours (total energy and alcohol intake, LTPA and TV time) in a cross-sectional Australian cohort. In this study, the sum of all health behaviours explained 27% and 45% of the observed educational inequalities in waist circumference for men and women, respectively. In addition, a smaller study of women [12] found dietary differences (a higher consumption of protein, carbohydrate and sucrose and a lower consumption of alcohol among those of the lowest SEP) were responsible for 40% of the relationship between the Hollingshead Index of social position and BMI. Total fibre, carbohydrate and sucrose consumption were the most important dietary mediators in the study [12]. Further, a study by Molarius *et al.*, [10] found that a greater frequency of heavy alcohol use and sedentary behaviour (TV watching time) among men of the lowest education group explained 22% of the association between education and a continuous measure of BMI. Despite the different behaviours used, the overall magnitude explained was similar to our results.

Conversely, in the same study, a greater frequency of sedentary time, heavy alcohol use and regimented attitudes towards dietary fats among women of a lower SEP explained only 12% of the association between education and BMI [10]. Further, a study using the Ontario Food Survey [13] found health behaviours (fruit and vegetable intake and LTPA) were unable to account for a significant portion of the difference in BMI between high and low education or income groups [13]. The discrepancy between these two studies and ours is likely to be attributable to the differences in the definition and type of health behaviours investigated as well as the nature of the study populations.

Our finding that sweet snacks were inversely related to the socioeconomic inequalities in BMI was surprising, but may serve to reinforce the suggestion of Mozaffarian & Darmon [5,9] that the types of sweet food consumed vary considerably across social strata, have specific relationships with body weight and hence contribute uniquely to socioeconomic inequalities in BMI. Our results indicate that in future analyses, sweet foods should be combined with caution.

Our study has a number of strengths. These include a large sample size and thus the power to stratify by both SEP and sex, as well as measured height and weight and an extensive and validated FFQ [21,22]. Hence, we have been able to investigate the role of a wide spectrum of dietary factors and identify nuances in the sex-specific dietary factors that mediate the observed socioeconomic inequalities in BMI. For example, to decrease socioeconomic inequalities in obesity, focus should perhaps be directed at promoting the consumption of healthy savoury foods in place of unhealthy savoury foods, with less focus on meeting fruit and vegetable guidelines.

However, while our investigation of dietary measures was more comprehensive than previous studies, we do not explain substantially more of the socioeconomic inequalities in BMI. This raises the question of whether we require a better measure of diet to fully elucidate its mediating role, or further identification and investigation of the role of other factors driving susceptibility to weight gain among those of a lower SEP.

The remaining inequalities in BMI between high and low educational groups, after accounting for confounders and a wide range of dietary factors and LTPA, may be partly explained by factors that we have not taken into account in our study. These may include further individual level behaviours, such as a higher prevalence of sitting during leisure time [8], particularly among women [10], other reproductive factors [12], psychosocial stress [12], early life factors [23], self control [24], time perspective [25], sleep duration and quitting smoking within the previous four years [9], as well as a range of environmental factors, none of which were able to be investigated in our study. In addition, the inherent bias associated with self-reported variables may hinder our ability to ever entirely elucidate the mediating role of the health behaviours. In this study we do not attempt to account for upstream social determinants of health. To ultimately reduce socioeconomic inequalities in obesity, it is important that any public policy which intends to improve individual behavioural choices is implemented alongside policy which intends to resolve fundamental social and economic barriers to optimal health.

The primary limitation of our study is our use of cross-sectional survey data, limiting our ability to draw causal inferences for the relationships examined [19]. We found that the consumption of diet soft drink was associated with a higher BMI and whilst there is some support for this in the literature [26], this likely to be due to substitution of soft drink with diet alternatives among those with an already high BMI [9]. When assessing this relationship longitudinally diet soft drink is generally associated with weight loss over time [9,27]. However, due to the contemporary and emerging mediation methods utilised and the difficulty in obtaining large data sets with measured height and weight and a rich source of diet and physical activity information, we believe it was appropriate to first explore these questions with the cross-sectional data we had available. Although it was encouraging that our overall mediation results were robust in sensitivity analyses, it will however be essential that they are confirmed in prospective analyses in the future.

We are also limited by the single measure of SEP used in this study and it is possible that other indicators of SEP may yield different results, as different measures of SEP have been differentially associated with BMI and health behaviours. It is important that mediators of the relationship between other measures of SEP and BMI are examined [2], as well as a more detailed analysis of the role of highest educational attainment. Further limitations include our use of self-reported health behaviours, which may introduce a social-desirability bias, the low levels of consumption of diet soft drink and soft drink within the study population and the voluntary nature of the MCCS study population, which may limit the generalisability of our results to the general population. In particular the greater percentage of women and our exclusion of Southern European born participants demonstrates the selected nature of our population*.* However, there is no reason to believe the internal relationships between SEP, health behaviours and BMI are unique to the MCCS study participants and they are likely to be generalisable to a broader population. The most likely repercussion of each of these limitations is that we will have underestimated the effect of diet and LTPA on the relationship between education and BMI.

## Conclusions

Our results suggest that the lower frequency and intensity of LTPA and the higher consumption of unhealthy savoury foods among those of a lowest educational attainment are likely to be important contributors to the socioeconomic disparities observed in BMI in Australian men and women. Among women of a lower SEP, a higher diet soft drink consumption and lower alcohol consumption are also important. It is vital that effective and targeted public policy be implemented to support individuals in choosing more healthful behaviours to ultimately reduce the socioeconomic inequalities in obesity. Healthy choices should be the easy choices for all individuals across socioeconomic strata. Hence, it is important that future research examine the role of health behaviours, preferably objectively measured, in driving the socioeconomic inequalities in BMI using a longitudinal study, and include other possible drivers, such as sedentary time, to determine causal mediators of this relationship. This further research would facilitate the development of effective and targeted public policy to support individuals to choose more healthful behaviours and ultimately reduce the socioeconomic inequalities in obesity.

## Abbreviations

SEP: Socioeconomic position; BMI: Body mass index (kg/m^2^); LTPA: Leisure time physical activity; MCCS: Melbourne collaborative cohort study; FFQ: Food frequency questionnaire.

## Competing interests

The authors declare that they have no competing interests.

## Authors’ contributions

The authors’ responsibilities were as follows – AP and KB designed the research, AH provided essential materials, AP, KB, EG and AH contributed to the conceptualization and interpretation of the results, EG analysed the data, KB and EG co-wrote the manuscript. All authors had primary responsibility for the final content; all authors read and approved the final content.

## Pre-publication history

The pre-publication history for this paper can be accessed here:

http://www.biomedcentral.com/1471-2458/13/1214/prepub
